# Association of Low Lead Levels with Behavioral Problems and Executive Function Deficits in Schoolers from Montevideo, Uruguay

**DOI:** 10.3390/ijerph15122735

**Published:** 2018-12-04

**Authors:** Gabriel Barg, Mónica Daleiro, Elena I. Queirolo, Julia Ravenscroft, Nelly Mañay, Fabiana Peregalli, Katarzyna Kordas

**Affiliations:** 1 Department of Neurocognition, Catholic University of Uruguay, 11600 Montevideo, Uruguay; mdaleiro@hotmail.com (M.D.); elenaiqueirolo@gmail.com (E.I.Q.); fperegalli@gmail.com (F.P.); 2 School of Public Health and Health Professions, University of Buffalo, Buffalo, NY 14214, USA; juliarav@buffalo.edu (J.R.); kkordas@buffalo.edu (K.K.); 3 Faculty of Chemistry, University of the Republic of Uruguay, 11800 Montevideo, Uruguay; nellymanay@gmail.com

**Keywords:** lead exposure, child’s behavior, ADHD, executive functions

## Abstract

The negative effect of lead exposure on children’s intelligence is well-documented. Less is known about the impact of lead on the use of executive functions to self-regulate behavior. We measured blood lead level (BLL) in a sample of first grade children from Montevideo, Uruguay (n = 206, age 6.7 ± 0.5 years, 59.7% boys). Behavior was assessed with teacher versions of the Conners Rating Scale (CRS) and the Behavior Rating Inventory of Executive Functions (BRIEF). Mean BLL was 4.2 ± 2.1 μg/dL; 10% had mild-to-severe ratings of Attentional Deficit with Hyperactivity Disorder (ADHD) (T score > 65). In negative binomial regression, BLL was not associated with CRS sub-scales, but was associated with a poorer ability to inhibit inappropriate behaviors, prevalence ratio (PR) [95% CI]: 1.01 [1.00, 1.03] as measured by the BRIEF. In covariate-adjusted models, the association with BLL was attenuated. When stratified by sex, the covariate-adjusted association between BLL, hyperactivity, poorer inhitibion, emotional control, and behavioral regulation was marginally significant for girls but not boys. In summary, among children with low lead-exposure, we found some, but nonetheless modest, evidence of a relationship between higher BLL and child behavior. If confirmed by larger studies and other objective measures of behavior, such links could have implications for learning and social interaction, particularly among girls.

## 1. Introduction

Lead exposure, even at low levels, has been associated with attention deficit hyperactivity disorder (ADHD) and other disruptive behaviors in school children. In their review, Eubig, Aguiar and Schantz [1] found this association with blood lead levels (BLL) below 10 µg/dL, although there is evidence for an association below 5 µg/dL [2,3]. Lead exposure and its effects on behavior take place in the context of other environmental and individual factors [4]. One such factor is the child’s neuro-cognitive development, especially the maturation of executive functions (EF), which is crucial to the self-regulation of behavior.

Considering the range of links between BLL and behavior problems, as well as alterations in cognitive function, including lower IQ, learning disabilities, attentional problems, and working memory deficits [5,6,7,8,9,10,11], Nigg et al. [12] proposed that weak cognitive control may mediate the effect of lead exposure on behavior, but found in their study that an effect of lead that was independent of IQ. Eubig et al. [1] showed that deficits in EF related to lead are similar to those associated with ADHD. Very few studies have examined ADHD symptoms in lead-exposed children together with a wide profile of executive functions, particularly those related to emotion regulation [13,14,15].

In the brain, lead exposure has been associated with a deficit in the functioning of dopaminergic neurotransmission, especially in the mesocorticospinal circuit [16]. The functioning of this system has been linked with motivation [17], and as such, it contributes to regulating behavior. Moreover, dopaminergic dysregulation associated with lead also affects areas of the prefrontal cortex [18], which have been linked with impairment of EFs typically observed in children with ADHD, including working memory, cognitive flexibility, and regulation of behavior [19,20,21].

Sex, either through biological differences or gender-typical behaviors, may affect patterns of toxicant exposure and toxicokinetics [22]. Despite this, studies that consider sex in the collection and analysis of neurobehavioral data in relation to toxicant exposure are scarce, and mostly use animal models [23,24]. Therefore, it is important to study the potential moderation by sex in the association between BLL and behavior in settings where children are chronically exposed to contaminants. Interestingly, a recent review [25] concluded that lead exposure produces a consistent gender-specific neurotoxic effect in the cognitive domain, with males being more affected. On the other hand, the same review observed that the effect of BLL on behavior was similar for boys and girls.

Behavior in early school years is critical to the formation of early teacher perceptions of children’s personality or skills [26]. In turn, these perceptions exert a powerful influence on academic achievement [27,28,29]. Moreover, gender stereotypes related to children’s behavior also interact with other beliefs as teachers develop their attitudes toward their students [30]. In low and middle-income countries (LMICs), where teacher and parent expectations regarding classroom behavior of boys and girls are strong, sex differences may be particularly important to investigate [31,32,33].

Considering that lead affects the neural basis of EF, it is reasonable to expect that children with higher BLL will present with EF impairments. On the other hand, lead neurotoxicity produces disruptive behavioral symptoms. Consequently, lead exposure could affect children’s self-regulation capacity, a key component for social and academic development. To date, these effects have not been investigated in the same group of children, accounting for potential sex differences and relatively low-level exposure to lead. We hypothesized that children with elevated BLLs would have higher scores on ADHD-related behaviors and greater deficits in executive functions generally, and more specifically in those relevant to behavioral regulation (inhibition, shifting, and emotional control) [34].

## 2. Materials and Methods

### 2.1. Study Setting

The study was conducted in Montevideo, the capital of Uruguay. Montevideo has several industries, including an oil refinery, and is crisscrossed by several major motorways, which are heavily traveled. Although leaded gasoline has been phased out of use in 2004, lead remains in the environment and other pollutants are a problem as well. Previous studies have shown that children in Montevideo are exposed to several toxic metals including lead, arsenic, cadmium, and manganese [35,36]. The present study was carried out in private elementary schools in the several neighborhoods of Montevideo, which were considered at risk for metal exposure based on previous epidemiological studies or knowledge of the pediatric population served by the Clinic for Environmental Contaminants, at the Pereira Rossell Hospital for Children (the national reference treatment center for lead poisoning).

### 2.2. Participant Recruitment

A detailed protocol for the study, including recruitment, has been provided elsewhere [37,38]. Briefly, directors of the schools located in the neighborhoods of interest were contacted to have an interview and obtain permission to invite parents to an informational meeting. At the meeting, the rationale and procedures of the study (including risks, benefits, and duration) were explained. All first-grade children who regularly attended the participating schools were eligible to participate. The sole exclusion criterion was a previous diagnosis of lead poisoning (defined as BLL > 45 µg/dL), which would have necessitated medical treatment. None of the children were excluded based on this criterion. Altogether, 357 children and their mothers agreed to participate in the study. Of those, 206 had records for all the variables of interest (exposure, outcome, and covariates) and were included in a complete-case analysis.

Participant teachers and parents signed an informed consent form after study personnel clarified all their questions. The protocol of the study was approved by the Ethics Committee for Research Involving Human Participants at the Catholic University of Uruguay (No. B041108), the Ethics Committee of the Faculty of Chemistry at the University of the Republic of Uruguay, the Office of Research Protections at the Pennsylvania State University, and the Institutional Review Board at the University at Buffalo (No. B251111).

### 2.3. Assessments

#### 2.3.1. Anthropometric Measurements

Children’s height was measured in triplicate to the nearest 0.1 cm, using a portable stadiometer (Seca 214, Shorr Productions, Colombia, MD, USA). Children were weighed in triplicate to the nearest 0.1 kg using a digital scale (Seca 872, Shorr Productions, Colombia, MD, USA). Children wore light clothing but no shoes, sweaters, or jackets. The measurements were combined to calculate the body mass index (BMI), as kg/m^2^.

#### 2.3.2. Blood Lead Analysis

Fasting blood was collected by a phlebotomy nurse at the school during a morning visit (in the presence of the child’s mother) between 8 and 11 am. Approximately 3 mL of venous blood was collected from each child using a 25-gauge safety butterfly blood collection set (Vacutainer, Becton Dickinson, Franklin Lakes, NJ, USA) in heparin coated trace metal free tubes (Vacutainer, Becton Dickinson, Franklin Lakes, NJ, USA) for lead analysis. An additional 3 mL venous blood was drawn into a serum tube with clot activator and separator gel (Becton Dickinson, Franklin Lakes, NJ, USA). The serum tubes were left to stand for 45 min, then centrifuged for 10 min at 3000 rpm. Approximately, 250 µL of serum was aliquoted for serum ferritin (SF) and C-reactive protein measurements.

Whole blood was stored on ice and then taken to the Toxicology Laboratory at the Faculty of Chemistry at the University of the Republic (CEQUIMTOX) in Montevideo, Uruguay for analysis. Blood lead concentrations were measured using atomic absorption spectrometry (AAS, VARIAN SpectrAA-55B, Agilent Technologies, Santa Clara, CA, USA) via flame or graphite furnace ionization techniques, depending on the volume of whole blood available. The graphite furnace was used in those blood samples for which volume was below 2 mL, and therefore insufficient for a flame furnace. The detection limit was 1.8 µg/dL for the flame and 0.8 µg/dL for graphite furnace AAS techniques.

#### 2.3.3. Serum Ferritin Analysis

Serum samples were shipped on dry ice to the Department of Nutritional Sciences, Pennsylvania State University to be stored at −20 °C until analysis. SF concentrations were determined in duplicate by an immunoradiometric assay (Coat-A-Count Ferritin IRMA; SIEMENS Diagnostic Products, Tarrytown, NY, USA) or an enzyme immunoassay (Spectro Ferritin, RAMCO Laboratories, Stafford, TX, USA). The enzyme-linked immunosorbent assay (ELISA) was used when the laboratory no longer had the capability to handle radioactive materials. The samples were allowed to come to room temperature and mixed gently by swirling before use in the assay. An aliquot of 10 µL was used for the analysis. The intra-assay and inter-assay coefficients were 4.2% and 9.5%, respectively, for the IRMA method and 1.7% and 7.6%, respectively, for the ELISA method. The use of different assays was addressed by deriving a correction factor, with the IRMA method serving as gold standard, and both values being log-transformed prior to the derivation step, and back-transformed for the main analysis.

Concentrations of C-reactive protein (CRP) were measured to identify the presence of subclinical inflammation/infection in the study children. CRP was analyzed in duplicate using an ELISA technique described by Erherdt and colleagues [36,37,38]. Serum control samples (Liquicheck, Bio-Rad, Hercules, CA, USA) were used as standards. Intra-assay and inter-assay CVs were 4.9% and 8%, respectively. To determine iron status, CRP-adjusted serum ferritin (SF) values were used because inflammation affects iron storage.

#### 2.3.4. Parental Questionnaires

Caregivers completed a questionnaire about socio-demographic characteristics of the family. They were asked about the child’s medical history and home environment, the possession of 12 household items (TV, video player, DVD player, computer, video games, radio, sound equipment, refrigerator, washer, home phone, cellular phone, and car). A possessions index was computed based on a factor analysis of the responses and consisted of five items: computer, car, freezer, washing machine, and landline phone. These items were summed to create an index score with a range of values between 0 and 5. The questionnaire also collected information about maternal education and employment. It was self-administered but research staff were on hand to provide assistance.

#### 2.3.5. HOME Inventory

The Home Observation for Measurement of the Environment (HOME) Inventory [39] was used to assess the quality of the children’s home environments. The inventory contains 59 items grouped into 8 scales: parental responsibility, encouraging maturity, emotional climate, learning materials and opportunities, active stimulation, family participation, parental involvement, and physical involvement. It allows calculation of a global score. Higher values of the score mean better rearing environment. The HOME Inventory was administered by a social worker who visited the child’s home at a previously scheduled time.

#### 2.3.6. Child’s IQ

The general intelligence ability (GIA) of the child was measured through the Woodcock-Muñoz battery (Riverside Publishing, Rolling Meadows, IL, USA), a paper-and-pencil test that assesses a wide range of cognitive skills and their use, based on the Cattell–Horn–Carrol theory of intelligence and validated for Spanish-speaking populations [40]. The GIA is a global standardized score, equivalent to Weschler’s IQ. The GIA score is standardized on a 500 base (W score) and is calculated from the results of the following sub-tests: verbal comprehension, concept formation, numbers reversed, visual-auditory learning, spatial relations, sound blending, and visual matching.

#### 2.3.7. Behavior Ratings of the Child by Teachers

The Conners Rating Scales-Revised (CRS-R, MHS, North Tonawanda, NY, USA) short form for teachers was used to measure the presence of hyperactive, oppositional, cognitive, and ADHD-like behaviors. The CRS-R is a standardized instrument based on a large normative sample of children and adolescents from the U.S. and Canada, including Spanish-speaking children, which is specifically relevant to the Spanish version of the instrument. The revised scales are based on updated, DSM-V, criteria for ADHD. The scales consist of 28 statements that describe typical behaviors or situations for children attending school. The teachers are asked to think of each child’s behavior in the previous 1 month and indicate whether it occurs never or rarely (assigned a score of 0), occasionally (score of 1), frequently (score of 2), or with high frequency (score of 3). The types of behaviors queried include “inattentive, easily distracted,” “is always moving or acts as if pushed/propelled by a motor,” “leaves his seat in the classroom when he is expected to be seated,” “does not have any interest in school work,” “has difficulty playing or entertaining himself without making noise,” etc. The questions are split up into four exclusive scales: hyperactive, oppositional, cognitive problems, and an ADHD index. Answers to the questions in each scale are summed and the raw scores are converted into T scores based on age-and-sex appropriate reference groups. Higher scores represent more problems and a T-score above 70 on any of the scales is considered to represent clinically significant behavior problems.

The Behavior Inventory of Executive Function (BRIEF, PAR Inc, Lutz, FL, USA) is an instrument consisting of 86 statements on child behavior, such as “overreacts to small problems,” “when given three things to do, remembers only the first or last,” “forgets to hand in homework, even when completed,” “resists change of routine, foods, places,” “thinks too much about the same topic.” Teachers are asked to think about each child’s behavior in the previous 6 months and to indicate whether the behavior is never (scored as 0), sometimes (scored as 1), or often (scored as 2) a problem. The BRIEF is scored as eight non-overlapping scales: inhibit, shift, emotional control (all falling under the behavioral regulation index), and initiate, working memory, plan/organize, organization of materials, and monitor (falling under the metacognition index). A global executive composite score is also derived. All raw scores obtained by summing item responses are converted to T-scores, interpreted in the same way as for Conners Scales. BRIEF is based on a large normative sample and the Spanish translation is provided by the publisher. The questionnaires have a robust correlation with “gold standard” measures of children’s behavior [41,42], as well as with parental reports [43]. Although both the Conners and BRIEF are multi-informant instruments, for reasons of economy of the participant’s time, only the teacher questionnaires were administered.

#### 2.3.8. Statistical Methods

##### Complete-Case Analysis

All statistical analyses were performed using STATA 12.0 (STATA Corp., College Station, TX, USA). Cases with incomplete records on any variable of interest were excluded from analysis (n = 147). Complete-cases and excluded cases were compared on these variables using a *t*-test (for continuous variables) or χ^2^ (for categorical variables). The exposure variable was untransformed BLL because it approximated a normal distribution. The outcome variables included the standardized scores (T) for the four Conners scales (hyperactivity, oppositional, cognitive problems/inattention, and ADHD index) and the eight BRIEF scales (inhibit, shift, emotional control, initiate, organization of materials, working memory, plan/organize, and monitor). The derived scores from the BRIEF scales were also included as outcome variables: behavioral regulation index, metacognition index, and global executive composite.

Due to the positively-skewed distribution of behavioral scores, their range (40–90 points), and overdispersion (variance larger than mean), the association between BLL and each behavioral/EF scale score was modeled using negative binomial regression. Negative binomial regression uses the log link function to model the expected log count of the dependent variable for a given change in the indepdenent variable (or percent difference in the dependent variable when the regression coefficients are exponentiated). Because our dependent variable was measured in a cross-sectional study, we take the exponentiated coefficient to represent a prevalence ratio of higher scores. In our modeling strategy, BLL was first modeled as a continuous variable. Next, BLL was dichotomized at 5 μg/dL, the current actionable level set by the U.S. Centers for Disease Control and Prevention. The difference in behavior/EF scale scores was estimated for children <5 and ≥5 μg/dL, in unadjusted and covariate-adjusted regressions. Potential confounders were identified using a directed acyclic graph (DAG) [44]. The variables chosen for consideration in the DAG were based on existing literature on the effects of lead exposure in behavior and/or executive functions [3,11,12,13,16]. The final model covariates seleted by this method included child IQ (GIA score), iron status (continuous), body mass index (continuous), household possessions (continuous), maternal education (years, continuous), and current parent smoking (yes/no). The inclusion of the HOME Inventory score was deemed unnecessary, given the use of the child’s IQ in the model. Since two methods of chemical analysis were used depending on the volume of the sample, the blood lead testing method was another covariate used in the models. Since girls had a higher mean of BLL than boys (girls = 4.4, boys = 3.9, *t*-test *p* < 0.05) and there are differences in behavior between the sexes, in secondary analyses, linear regression models with BLL as continuous variable were stratified by sex.

##### Multiple Imputation and Modeling

Due to missing data on a number of variables, including exposure, outcome, and covariates, we carried out a multiple imputation analysis. Multiple imputation (MI) requires that the data meet the assumption of missing completely at random (MCAR) or missing at random (MAR). The majority of the missing data in our study were in the behavior ratings by teacher. We cannot assume MCAR. However, we know that the reason teachers did not complete ratings had to do with how busy they were, and not with the children’s characteristics; we found, for example, that the children with and without behavior ratings did not differ on BLL or IQ. The imputation model consisted of all the variables in the final analytical model (BRIEF variables, Conners variables, child IQ, sex, age, blood lead (both continuous and categorical), blood lead testing method, HOME score, iron status, maternal education, parental smoking, possessions, and BMI). Additionally, maternal occupation, household density/crowding, and a lead by sex interaction term were entered. We implemented the chained imputation command in STATA, specifying that 50 imputations be performed and using the default number of cycles of regression switching. Variables were modeled according to their distribution using the *regress, logit, ologit*, and *poisson* functions. Predictive mean matching imputation (*ppm*) was utilized to maintain the non-normal distribution of the behavior rating scales, and the discrete nature of the T-scores. Following a recommendation of Morris and collegues [45], 10 donors were specified in the STATA *pmm* command. Once the imputed datasets were estimated, we re-ran the analytical models described above using STATA’s *mi estimate*, which uses Rubin’s rules to combine the beta coefficients and 95% CIs into a single estimated value. In order to obtain gender stratified estimates, the previous imputation and analytical model were re-run, stratified by gender using STATA’s *by* sub-command.

## 3. Results

### 3.1. Sample Characteristics

A number of variables had missing observations (Supplemental Table S1) and children with missing data (n = 147) were excluded from analysis. The complete-case sample consisted of 206 children; their sociodemographic, biological, cognitive, and behavioral characteristics are shown in Table 1. Those children who were excluded from the analysis had similar characteristics to the complete-case sample, with the exception of sex and mother’s occupation. The excluded group had more girls and fewer unemployed mothers.

The study sample had a mean age of 81 months (standard deviation/SD = 6.6), a mean BLL of 4.2 µg/dL (SD = 2.1), and 41.7% presented iron deficiency, defined as CRP-adjusted serum ferritin below 15 ng/mL. Approximately a third of the mothers were unemployed. The Woodcock–Muñoz GIA scores had a mean of 472 (SD = 11.7), which is within the expected range for children aged 6–8 years. Mean sub-scale T scores on the Conners were all approximately 50 points, with wide variance.

The mean T scores on the BRIEF sub-scales were also relatively low, with the exception of initiate, working memory, and plan/organize (T scores of 58, 57, and 57, with SD of 14.6, 14.7 and 14.7 respectively). The prevalence of clinical behavior problems (T score ≥ 70) was generally modest but there were some sex differences (Supplemental Table S2). Notably, 17.7% of girls had a T score ≥70 in the ADHD Index of the Conners while the prevalence was 8.85 among boys (*p* = 0.026). Also, on the inhibition scale of the BRIEF, 16.7% of girls and 8.1% of boys had a T score of ≥70 (*p* = 0.028).

### 3.2. Association between BLL and Mean Behavior Rating Scores

The association of BLL with behavior rating scores was tested through negative binomial models, a separate model run for each of the Connners and BRIEF scores (Table 2). There were no statistically-significant associations between the BLL and teacher ratings of behavior using Conners scales. In an unadjusted model, BLL was associated with 1% higher prevalence ratio on the inhibit sub-scale of the BRIEF for each 1 µg/dL higher BLL (95% CI: [1.00, 1.03], *p* < 0.05).

After adjusting for covariates, the association became somewhat attenuated but was consistent with the bivariate model in that higher BLLs were related to higher mean scores (1.01 [1.00, 1.03], *p* < 0.1). BLL was also associated with higher PR on the behavioral regulation index in unadjusted analysis (1.01 [1.00, 1.03], *p* < 0.1), but this became attenuated after covariate control. Analyses conducted with imputed data yielded similar estimates (Supplemental Table S3).

The association between BLL ≥ 5 µg/dL and the prevalence ratio of behavior ratings is shown in Table 3. On both the Conners and the BRIEF scales, children with BLL ≥ 5 µg/dL had slightly higher PRs (2–4% in unadjusted models 1–3% in covariate-adjusted models) than children with BLL < 5 µg/dL, but none of these associations were statistically significant. Analysis of imputed data yielded similar findings (Supplemental Table S4).

### 3.3. Association between BLL and Behavior Ratings by Teachers, Stratified by Sex

The relationship between BLL and the PR of behavior ratings on the Conners and BRIEF in relation to BLL were examined separately for boys and girls, and only the covariate-adjusted models are presented in Table 4. Each 1 µg/dL in BLL was associated with 2% higher PR (*p* < 0.1) on Conners hyperactivity sub-scale among girls but not boys. On the BRIEF, girls with higher BLL had higher PR on the inhibit (1.02 [1.00, 1.05]), emotional control (1.02 [1.00, 1.05]) scales, and the behavioral regulation index (1.03 [1.00, 1.05]). There were no associations among boys, but it is important to point out that the confidence intervals overlapped.

Models conducted with imputed data produced similar findings (Supplemental Table S5). In addition to the findings from the complete-case analysis, BLL in the imputed models was associated with higher T scores on the emotional control (1.02 [1.00, 1.05], *p* < 0.1) scale of the BRIEF among girls.

## 4. Discussion

The objective of this study was to investigate the association of BLL and behavior in a sample of 6–8-year-old children. Although few findings reached statistical significance, likely due to the small sample size and the relatively low levels of BLL, some interesting patterns emerged. First, children with higher BLLs had a higher likelihood to receive higher scores on the inhibit scale, indicating a poorer ability to inhibit inappropriate behaviors, a key component of self-regulation. Second, BLL appeared to be associated, albeit very modestly, with hyperactivity, poorer inhibition, and emotional control in girls but not boys. Although biological factors could mediate these result patterns, a gender bias in teacher perception and expectation of classroom behavior may also explain these findings. We consider this further below.

BLL has been consistently associated with ADHD [3,6,7,8,9,10]. According to Nigg and Casey [46], the association is stronger with the hyperactivity–impulsivity factor than the inattention–disorganization factor of the ADHD. The former is related to failures in bottom-up aspects of cognitive control such as prediction of rewards and inhibition of inappropriate responses. This mechanism relies on the dopaminergic projections between the prefrontal cortex and the striatum, a proposed target for lead toxicity in the brain [47,48,49]. Executive functions, and particularly the ability to inhibit prepotent responses, have been found to mediate the relationship between BLL and hyperactivity–impulsivity [12]. Although this effect of BLL is well-documented in animal models, findings in children are inconsistent [1]. One problem is that the evidence is based mainly on continuous performance tasks of attention in laboratory settings, while problems with response inhibition are better observed in real-life settings, like the classroom. Conventional experimental tasks demand relatively simple responses to single events while learning environments are full of simultaneous stimuli which requires a series of complex responses, including rapid selection or inhibition [50]. Observational instruments of the child in the natural environment are more adequate to measure those problems. Nevertheless, few studies assess the effect of BLL in executive functions through children’s behavior in learning and social situations. In line with our results, a study of 3–7-year-old children from Chennai, India [11], using the same instrument as in the present research (the BRIEF), found stronger associations between BLL and EF than between BLL and ADHD index, anxiety, or sociability.

One finding with regard to the prevalence of behavior problems is worth mentioning. For some of the behavioral measures, namely inhibition and ADHD index, we found higher prevalence of clinically-significant problems among girls than boys. This is contrary to previous evidence. A study conducted in 31 countries with children aged 6 to 16 years showed that externalizing problems are more frequent in boys than girls [51]. Several meta-analyses also found that ADHD is more prevalent in boys than in girls, as well as the severity of symptoms such as impulsivity, hyperactivity, and oppositional behavior [52,53,54,55]. A meta-analysis about gender differences in continuous performance tasks among children with ADHD concluded that boys were more impulsive than girls, but there were no differences in inattention problems [56]. In the classroom, boys with ADHD are more impulsive, aggressive, and hyperactive than girls with ADHD, but this difference is not significant in children with sub-clinical behavioral problems [57].

The fact that behavioral problems are more frequent in boys than girls makes it difficult to measure the role of sex in the association between lead exposure and behavior. Neuropsychological and electrophysiological findings suggest that the executive function impairment, especially response inhibition, is a correlate of ADHD, independent of age and gender [58,59,60]. A study using a representative sample of U.S. children from the U.S. National Health and Nutrition Examination Survey (NHANES) 1999–2002 found a consistent relation between BLL and ADHD symptoms (more prevalent in boys), but failed to find a significant interaction between BLL and gender [61]. Data from the Canadian Health Measures Survey 2007–2009 [6], from a longitudinal study in Korea [62], and from prenatal exposure in Belgium [63] also show a higher prevalence of behavioral problems in boys but not a higher effect of BLL in boys than girls. In our study, the association of BLL with behavioral problem was stronger in girls.

Although distinguishing sex differences is crucial for understanding the toxicological response at a biological level, very few studies report results in a way that allows for quantifying gender-based exposures and outcomes. Specifically, there is little information on environmental exposures that affect girls differently compared to boys [22]. A study about gender differences in mixed metal exposure, including lead, in Bangladesh concluded that women and children are at more risk of metal exposure, as well as micronutrient deficiency [24]. However, sex differences in toxicant-outcome effects can be also mediated by social factors.

In our study, the fact that BLL appears more strongly associated with behavioral ratings among girls than boys could be explained by teacher expectations and perceptions of girls‘ and boys’ behavior. Teacher expectations and beliefs about their students have a powerful influence on children’s academic achievement more than 10 years later [27]. This phenomenon was denominated the Pygmalion effect [64], and has received strong empirical support [28,29,65,66]. Sex is one of the factors that mediate teacher expectations of academic performance, IQ, or personality traits [67]. A meta-analytic review concluded that teachers tend to initiate more interactions, both positive and negative, with boys than with girls in the classroom [30]. In this context, lead exposure could have the same biological contribution to disruptive behavior in girls and boys, but may be more noticeable to teachers when it occurs in girls. The existence of a gender bias in teacher perceptions of behavior is more likely in cultural settings with rigid gender roles, like South America [31,33]. For example, in Brazil, a similar pattern of behavioral effects prevalent in girls was found in relation to manganese exposure [68]. In that sense, teacher and parent expectations and perceptions could amplify the negative effect of lead exposure on behavior for specifically for girls.

Our findings should be interpreted in light of study strengths, which include the examination of several aspects of children’s behavior, not only impulsivity/hyperactivity and attentional problems, but also a wide range of executive functions, in a naturalistic setting with low level of lead exposure. We also conducted gender-stratified analysis, given the growing recognition that the effects of environmental exposures may differ between the sexes, even at a young age. Another strength is that models were adjusted for a number of important contextual variables. At a social level, we included measures of the family environment (HOME inventory) as well as socio-economic status (index of household possessions and maternal education). The analysis also controlled for iron status because this metal shares a common route of absorption and neural targets with lead [16].

Nevertheless, several limitations need to be acknowledged. First, the study employed a reduced sample size, which limited statistical power. One reason for this was related to the teachers’ willingness to complete the questionnaires about their students, more so than any characteristics specific to the children. To compensate for this limitation, we performed multiple imputation models, and obtained similar results to the complete-case analysis, which suggests that the exclusion of participants based on missingness of behavior rating data did not result in a biased sample. Second, behavior was assessed only through teacher reports, without the inclusion of parents as informants. Both for the Conners and the BRIEF, teacher reports are considered the most objective and economical way to obtain information about children’s behavior, with an adequate correlation with parents reports and clinical interviews [41,42,43]. Nevertheless, because of potential biases in teacher expectations of behavior, studies in the future should include multiple informants and more objective measurements and/or clinical diagnoses. Finally, since this study is cross-sectional, it is not possible to make causal inferences about the effect of BLL in behavior and EF during child development. Prospective studies that measured lead exposure histories of children found a consistent association between BLL and lower IQ [69,70,71]. Therefore, longitudinal studies with larger samples that assess the effect of lead and other contaminants in the development of executive functions through infancy are required to understand the magnitude of impairment in self-regulation. These studies should include measures of teachers and family expectations of children’s behavior as mediator factors.

## 5. Conclusions

We found a pattern of modest associations that suggests relatively low-level lead exposure may modestly affect children’s executive functions that are important to emotional and behavioral regulation. Teachers expectations of classroom behavior in certain cultural settings may amplify the negative effect of lead on behavior for girls, but not boys.

## Figures and Tables

**Table 1:** Sample characteristics and comparison between children included and excluded from analysis due to missing data.

Variable	Complete-Case Analysis (*n* = 206)	*N*	Excluded from Analysis
	M ± SD or %		
Age (months)	81 ± 6.6	145	81 ± 6.1
Sex ^1,^*		147	
Male	59.7%		48.9%
Female	40.2%		51.0%
Blood lead level (μg/dL)	4.2 ± 2.1	109	3.9 ± 1.9
IQ (GIA score–Woodcock–Muñoz)	472 ± 11.7	133	473 ± 12.5
Serum ferritin <15 ng/mL (adjusted by CRP)	41.7%	98	34.6%
HOME score ^2^	44.1 ± 8.4	126	45.4 ± 7.2
Household possessions	3.4 ± 1.1	103	3.4 ± 1.1
Maternal education (years completed)	8.9 ± 2.7	128	9.3 ± 2.6
Either parent smokes	54.3%	100	51.0%
Mother’s occupation ^1,^*		119	
Unemployed	35.4%		21.8%
Employed	64.6%		78.2%
Body Mass Index	16.8 ± 2.6	120	16.9 ± 2.5
CTRS-R:S (T score) ^3^		82	
Oppositional	53.9 ± 13.9		52.5 ± 11.5
Cognitive Problems/Inattention	53.8 ± 12.5		55.1 ± 13.2
Hyperactivity	54.5 ± 11.8		53.0 ± 11.1
ADHD Index	54.4 ± 11.2		55.0 ± 11.6
BRIEF (T score) ^4^		79	
Inhibit	53.8 ± 12.1		52.7 ± 11.4
Shift	54.2 ± 11.4		54.9 ± 11.9
Emotional Control	55.4 ± 14.5		54.1 ± 13.5
Initiate	58.1 ± 14.6		59.1 ± 15.2
Organization of Materials	53.7 ± 11.5		54.1 ± 12.0
Working Memory	57.7 ± 14.7		59.4 ± 15.3
Plan/Organize	57.8 ± 14.7		60.2 ± 16.2
Monitor	56.0 ± 13.3		56.3 ± 13.8
Behavioral Regulation Index	55.0 ± 13.8		54.3 ± 12.3
Metacognition Index	57.4 ± 14.0		58.7 ± 14.5
Global Executive Composite	57.0 ± 13.7		57.6 ± 13.6

^1^*t*-test for continuous variables and χ^2^ for categorical variables were carried out to test differences between included and excluded population, * *p* < 0.05. ^2^ Home Observation for the Measurement of the Environment Inventory (HOME) contains 59 items with a total possible score of 59. ^3^ Conners Behavior Rating Scales for Teachers—Revised Short Form. ^4^ Behavior Rating Inventory of Executive Function for teachers.

**Table 2:** Association (prevalence ratio) between blood lead concentrations and behavior ratings by teachers among 5–8-year-old children from Montevideo, Uruguay (*n* = 206).

Behavior Rating Scale (T score)	Unadjusted	Covariate-Adjusted ^1^
	PR [95% CI]	PR [95% CI]
CTRS-R:S ^2^		
Oppositional	1.00 [0.99, 1.02]	1.00 [0.98, 1.02]
Cognitive Problems/Inattention	1.01 [0.99, 1.02]	1.01 [0.99, 1.02]
Hyperactivity	1.01 [0.99, 1.02]	1.01 [0.99, 1.02]
ADHD Index	1.01 [0.99, 1.02]	1.00 [0.99, 1.02]
BRIEF ^3^		
Inhibit	1.01 [1.00, 1.03] **	1.01 [1.00, 1.03] *
Shift	1.00 [0.99, 1.02]	1.00 [0.99, 1.01]
Emotional Control	1.01 [0.99, 1.03]	1.01 [0.99, 1.02]
Initiate	1.01 [0.99, 1.03]	1.01 [0.99, 1.02]
Organization of Materials	1.00 [0.98, 1.01]	0.99 [0.98, 1.01]
Working Memory	1.00 [0.98, 1.02]	1.00 [0.98, 1.01]
Plan/Organize	1.00 [0.99, 1.02]	1.00 [0.98, 1.01]
Monitor	1.01 [0.99, 1.02]	1.01 [0.99, 1.02]
Behavioral Regulation Index	1.01 [1.00, 1.03] *	1.01 [1.00, 1.03]
Metacognition Index	1.00 [1.00, 1.02]	1.00 [1.00, 1.02]
Global Executive Composite	1.01 [0.99, 1.02]	1.01 [0.99, 1.02]

^1^ Models adjusted for child IQ, child iron status, and body mass index, blood lead testing method, household possessions, maternal education, current parent smoking (yes/no). ^2^ CTRS-R:S = Conners Behavior Rating Scales for Teachers—Revised Short Form. ^3^ BRIEF = Behavior Rating Inventory of Executive Function for teachers. ** *p* < 0.05, * *p* < 0.1.

**Table 3:** Association (prevalence ratio) between blood lead concentrations and behavior ratings by teachers, comparing children with BLL ≥5 to those with <5 µg/dL (*n* = 206).

Behavior Rating Sub-Scale (T score)	Unadjusted	Covariate-Adjusted ^1^
	PR [95% CI]	PR [95% CI]
CTRS-R:S ^2^		
Oppositional	1.00 [0.93, 1.07]	0.98 [0.91, 1.06]
Cognitive Problems/Inattention	1.03 [0.96, 1.11]	1.02 [0.97, 1.08]
Hyperactivity	1.03 [0.96, 1.10]	1.01 [0.95, 1.08]
ADHD Index	1.02 [0.96, 1.09]	1.01 [0.95, 1.07]
BRIEF ^3^		
Inhibit	1.03 [0.97, 1.10]	1.02 [0.96, 1.09]
Shift	1.03 [0.97, 1.10]	1.02 [0.96, 1.08]
Emotional Control	1.04 [0.96, 1.12]	1.02 [0.95, 1.10]
Initiate	1.04 [0.97, 1.13]	1.03 [0.96, 1.10]
Organization of Materials	1.00 [0.94, 1.07]	0.99 [0.93, 1.05]
Working Memory	1.03 [0.96, 1.11]	1.02 [0.95, 1.09]
Plan/Organize	1.03 [0.96, 1.12]	1.02 [0.95, 1.09]
Monitor	1.02 [0.95, 1.10]	1.01 [0.94, 1.08]
Behavioral Regulation Index	1.05 [0.98, 1.13]	1.04 [0.97, 1.11]
Metacognition Index	1.03 [0.96, 1.11]	1.01 [0.95, 1.08]
Global Executive Composite	1.04 [0.97, 1.12]	1.02 [0.96, 1.09]

^1^ Adjusted for child IQ, child iron status and body mass index, blood lead testing method, household possessions, maternal education, current parent smoking (yes/no). ^2^ CTRS-R:S = Conners Behavior Rating Scales for Teachers—Revised Short Form. ^3^ BRIEF = Behavior Rating Inventory of Executive Function for teachers.

**Table 4:** Covariate-adjusted association (prevalence ratio) between blood lead concentrations and behavior ratings in boys and girls from Montevideo, Uruguay (*n* = 206).

Behavior Rating Sub-Scale (T Score)	Girls (n = 83)	Boys (n = 123)
	PR [95% CI] ^1^	PR [95% CI] ^1^
CTRS-R:S ^2^		
Oppositional	1.01 [0.99, 1.04]	0.99 [0.96, 1.02]
Cognitive Problems/Inattention	1.01 [0.99, 1.02]	1.01 [0.99, 1.03]
Hyperactivity	1.02 [1.00, 1.04] *	0.99 [0.97, 1.01]
ADHD Index	1.01 [0.99, 1.03]	1.00 [0.98, 1.02]
BRIEF ^3^		
Inhibit	1.02 [1.00, 1.05] *	1.00 [0.98, 1.01]
Shift	1.01 [0.99, 1.03]	0.99 [0.97, 1.01]
Emotional Control	1.02 [1.00, 1.05]	1.00 [0.97, 1.01]
Initiate	1.00 [0.98, 1.02]	1.00 [0.99, 1.03]
Organization of Materials	1.00 [0.98, 1.02]	0.99 [0.97, 1.01]
Working Memory	1.00 [0.98, 1.02]	1.00 [0.98, 1.02]
Plan/Organize	1.00 [0.97, 1.02]	1.00 [0.98, 1.02]
Monitor	1.01 [0.98, 1.03]	1.00 [0.98, 1.02]
Behavioral Regulation Index	1.03 [1.00, 1.05] *	0.99 [0.97, 1.01]
Metacognition Index	1.00 [0.98, 1.02]	1.00 [0.98, 1.02]
Global Executive Composite	1.01 [0.99, 1.04]	1.00 [0.98, 1.01]

^1^ Aadjusted for child IQ, child iron status, and body mass index, blood lead testing method, household possessions, maternal education, current parent smoking (yes/no). ^2^ CTRS-R:S = Conners Behavior Rating Scales for Teachers—Revised Short Form. ^3^ BRIEF = Behavior Rating Inventory of Executive Function for teachers. * *p* < 0.1.

## Supplementary Materials

The following are available online at http://www.mdpi.com/1660-4601/15/12/2735/s1. Table S1: Percent of children with behavioral problems (T score ≥65 or ≥70) stratified by gender. Table S2: Patterns of missing values in study variables. Table S3: Association between blood lead concentrations and behavior ratings by teachers among 5–8-year-old children from Montevideo, Uruguay using an imputed dataset (*n* = 353). Table S4: Covariate-adjusted association between blood lead concentrations and teacher ratings of behavior among 5–8-year-old girls and boys from Montevideo, Uruguay using an imputed dataset (*n* = 353). Table S5: Association between blood lead among 5–8-year-old children with blood lead concentrations <5 and ≥5 µg/dL using an imputed dataset (*n* = 353).
