# Supplementary material for: Association of Low Lead Levels with Behavioral Problems and Executive Function Deficits in Schoolers from Montevideo, Uruguay
Source: Int J Environ Res Public Health. 2018 Dec 4;15(12):2735. doi: 10.3390/ijerph15122735 (PMC6313712; doi:10.3390/ijerph15122735)
Supplement: Supplementary file 1 [file ijerph-15-02735-s001.zip › ijerph-388835-SI.pdf]

Int. J. Environ. Res. Public Health 2018, 15, 2735; doi:10.3390/ijerph15122735

Online Supplemental Materials

Supplemental Table 1. Patterns of missing values in study variables.

	Age	GIA score	Mat. education	HOME score	BMI	Mat. occupation	Blood lead level	Possessions	Crowding	Parent smoking	Serum ferritin	CTRS-R:S[1]	BRIEF[2]
Missing Values	2	14	19	21	27	28	38	44	47	47	49	65	68
Percent of sample with specific patterns of missing values[3]													
58%	1	1	1	1	1	1	1	1	1	1	1	1	1
10%	1	1	1	1	1	1	1	1	1	1	1	0	0
4%	1	1	1	1	1	1	1	0	0	0	1	1	1
3%	1	1	1	1	1	1	1	1	1	1	0	1	1
2%	1	1	1	1	0	1	0	1	1	1	0	1	1
1%	1	1	0	0	0	0	0	0	0	0	0	1	1
1%	1	1	1	1	1	0	1	0	0	0	1	1	1
1%	1	1	1	1	1	1	1	1	1	1	1	1	0
1%	1	1	1	0	1	1	1	1	1	1	1	1	1
1%	1	1	1	1	1	0	1	1	1	1	1	1	1
1%	1	1	1	1	1	1	0	1	1	1	0	1	1
1%	1	1	1	1	1	1	1	0	0	0	1	0	0
1%	1	1	1	1	1	1	1	1	1	1	0	0	0
All other patterns of missing values, <1% of sample													

[1]CTRS-R:S = Conners Behavior Rating Scales for Teachers—Revised Short Form; [2]BRIEF=Behavior Rating Inventory of Executive Function for teachers; [3]Values given as 1 = complete observations; 0 = missing observations.

Supplemental Table 2. Percent of children with behavioral problems (T score ≥ 65 or ≥70) stratified by gender

	Girls(%)	Boys(%)

CTRS-R:S[a]		
Oppositional		
T score ≥ 65	13.1	19.6
T score ≥ 70	13.1	14.6
Hyperactivity		
T score ≥ 65	20.0	22.1
T score ≥ 70	13.8	12.6
Cognitive probl./Inattention		
T score ≥ 65	22.3	20.9
T score ≥ 70	19.2	13.9
ADHD Index		
T score ≥ 65	23.1	20.8
T score ≥ 70	17.7	8.8
BRIEF[b]		
Inhibition		
T score ≥ 65	19.0	15.7
T score ≥ 70	16.7	8.1
Shift		
T score ≥ 65	18.2	16.9
T score ≥ 70	13.5	11.2
Emotional Control		
T score ≥ 65	21.4	23.2
T score ≥ 70	17.5	15.1
Initiate		
T score ≥ 65	38.1	29.5
T score ≥ 70	30.9	22.0
Organization of Materials		
T score ≥ 65	15.0	21.4
T score ≥ 70	7.1	13.2
Working Memory		
T score ≥ 65	31.7	35.8
T score ≥ 70	24.6	26.4
Plan/Organize		
T score ≥ 65	31.5	33.9
T score ≥ 70	23.0	28.3
Monitor		

T score ≥ 65	29.3	24.5
T score ≥ 70	17.4	15.1
Behavioral Regulation Index		
T score ≥ 65	18.2	18.2
T score ≥ 70	15.1	10.1
Metacognition Index		
T score ≥ 65	31.7	33.9
T score ≥ 70	23.8	25.7
Global Executive Composite		
T score ≥ 65	27.8	27.0
T score ≥ 70	22.2	17.6

[a]Conners Teacher Rating Scales- Revised Short Form (sub-scales T score). [b]Behavior Rating Inventory of Executive Function for teachers (sub-scales T score).

Supplemental Table 3. Association (prevalence ratio) between blood lead concentrations and behavior ratings by teachers among 5-8-year-old children from Montevideo, Uruguay using an imputed dataset (n=353).

	Unadjusted	Covariate-adjusted[1]
**Behavior rating scale (T score)**	**PR [95% CI]**	**PR [95% CI]**
CTRS-R:S[2]		
Oppositional	1.00 [0.99, 1.02]	1.00 [0.99, 1.01]
Cognitive Problems/Inattention	1.01 [0.99, 1.02]	1.00 [0.99, 1.01]
Hyperactivity	1.01 [1.00, 1.02]	1.01 [1.00, 1.02]
ADHD Index	1.01 [0.99, 1.02]	1.01 [1.00, 1.02]
BRIEF[3]		
Inhibit	1.01 [1.00, 1.02]*	1.01 [1.00, 1.02]
Shift	1.00 [0.99, 1.02]	1.00 [0.99, 1.01]
Emotional Control	1.01 [1.00, 1.02]	1.01 [1.00, 1.02]
Initiate	1.01 [0.99, 1.02]	1.01 [0.99, 1.02]
Organization of Materials	1.00 [1.00, 1.01]	1.00 [1.00, 1.01]
Working Memory	1.00 [0.99, 1.02]	1.01 [0.99, 1.01]
Plan/Organize	1.01 [0.99, 1.02]	1.01 [0.99, 1.01]
Monitor	1.01 [1.00, 1.02]	1.01 [1.00, 1.02]
Behavioral Regulation Index	1.01 [1.00, 1.03]*	1.01 [1.00, 1.02]
Metacognition Index	1.01 [0.99, 1.02]	1.00 [0.99, 1.02]
Global Executive Composite	1.01 [1.00, 1.02]	1.01 [1.00, 1.02]

[1]Models adjusted for child IQ, iron status, body mass index, blood lead testing method, household possessions, maternal education, and current parent smoking (yes/no). [2]CTRS-R:S = Conners Behavior Rating Scales for Teachers—Revised Short Form. [3]BRIEF = Behavior Rating Inventory of Executive Function for teachers. PR = Prevalence Ratio. *$p < 0.05$.

Supplemental Table 4. Association (prevalence ratio) between blood lead among 5-8-year-old children with blood lead concentrations <5 and ≥ 5 μg/dL using an imputed dataset (n=353).

	Unadjusted	Covariate-adjusted[1]
**Behavior rating scale (T score)**	**PR [95% CI]**	**PR [95% CI]**
CTRS-R:S[2]		
Oppositional	1.02 [0.95, 1.08]	1.00 [0.94, 1.06]
Cognitive Problems/Inattention	1.03 [0.97, 1.10]	1.02 [0.98, 1.07]
Hyperactivity	1.04 [0.99, 1.10]	1.04 [0.98, 1.09]
ADHD Index	1.03 [0.98, 1.09]	1.03 [0.98, 1.08]
BRIEF[3]		
Inhibit	1.04 [0.98, 1.09]	1.04 [0.98, 1.09]
Shift	1.04 [0.99, 1.10]	1.03 [0.98, 1.08]
Emotional Control	1.06 [0.99, 1.13]	1.05 [0.98, 1.12]
Initiate	1.04 [0.97, 1.11]	1.03 [0.97, 1.09]
Organization of Materials	1.01 [0.96, 1.07]	1.01 [0.95, 1.06]
Working Memory	1.03 [0.97, 1.11]	1.02 [0.97, 1.08]
Plan/Organize	1.02 [0.96, 1.09]	1.01 [0.95, 1.08]
Monitor	1.04 [0.98, 1.11]	1.03 [0.98, 1.09]
Behavioral Regulation Index	1.07 [1.00, 1.13]*	1.05 [0.99, 1.11]
Metacognition Index	1.03 [0.97, 1.10]	1.02 [0.97, 1.08]
Global Executive Composite	1.05 [0.99, 1.12]	1.04 [0.98, 1.10]

[1]Models adjusted for child IQ, iron status, body mass index, blood lead testing method, household possessions, maternal education, and current parent smoking (yes/no). [2]CTRS-R:S = Conners Behavior Rating Scales for Teachers—Revised Short Form. [3]BRIEF = Behavior Rating Inventory of Executive Function for teachers. PR = Prevalence Ratio. *$p < 0.05$.

Supplemental Table 5. Covariate-adjusted (prevalence ratio) association between blood lead concentrations and teacher ratings of behavior among 5-8-year-old girls and boys from Montevideo, Uruguay using an imputed dataset (n=353).

Behavior rating scale (T score)	Girls (n=158)	Boys (n=195)
	PR [95% CI][1]	PR [95% CI][1]
CTRS-R:S[2]		
Oppositional	1.01 [0.99, 1.03]	1.00 [0.97, 1.02]
Cognitive Problems/Inattention	1.01 [1.00, 1.03]	1.00 [0.99, 1.02]
Hyperactivity	1.02 [1.00, 1.04]*	1.00 [0.99, 1.02]
ADHD Index	1.01 [0.99, 1.03]	1.00 [0.99, 1.02]
BRIEF[3]		
Inhibit	1.02 [1.00, 1.04]*	1.00 [0.98, 1.01]
Shift	1.01 [1.00, 1.03]	1.00 [0.98, 1.01]
Emotional Control	1.02 [1.00, 1.05]*	1.00 [0.97, 1.01]
Initiate	1.01 [0.99, 1.03]	1.00 [0.99, 1.02]
Organization of Materials	1.00 [0.99, 1.02]	1.00 [0.98, 1.01]
Working Memory	1.00 [0.98, 1.02]	1.00 [0.99, 1.02]
Plan/Organize	1.00 [0.98, 1.02]	1.00 [0.98, 1.02]
Monitor	1.01 [0.99, 1.03]	1.00 [0.99, 1.02]
Behavioral Regulation Index	1.02 [1.01, 1.05]*	1.00 [0.98, 1.01]
Metacognition Index	1.01 [0.99, 1.02]	1.00 [0.97, 1.02]
Global Executive Composite	1.01 [1.00, 1.03]	1.00 [0.98, 1.01]

[1]Models adjusted for child IQ, iron status, body mass index, blood lead testing method, household possessions, maternal education, and current parent smoking (yes/no). [2]CTRS-R:S = Conners Behavior Rating Scales for Teachers—Revised Short Form. [3]BRIEF = Behavior Rating Inventory of Executive Function for teachers. PR = Prevalence Ratio. *$p < 0.05$.
